# Chronic myelogenous leukemia occurring in two brothers: The opposite sides of the same coin?

**DOI:** 10.1016/j.lrr.2021.100261

**Published:** 2021-07-31

**Authors:** Evgenia Verrou, Kyriaki Tsirou, Nikolaos Karampatzakis, Theodora Triantafyllou, Aggeliki Sevastoudi, Georgia I. Grigoriadou, Pavlina Konstantinidou, Panagiotis Panagiotidis, Eirini Katodritou

**Affiliations:** a Hematology Department, Theagenio Cancer Hospital, Thessaloniki, Greece; b Μolecular Diagnostics Unit, National and Kapodistrian University of Athens, Laikon General Hospital, Athens, Greece

**Keywords:** Familial CML, Tyrosine kinase inhibitors

## Abstract

Herein we present a rare case of two brothers diagnosed with CML four years apart. Importantly, our case of CML occurrence among siblings is the fifth one reported and the second one investigated by both, conventional cytogenetics and RT-PCR analysis. Moreover, although Ph chromosome was detected in both our patients, RT-PCR revealed the presence of two different BCR-ABL transcripts. Finally, both our patients have been followed for a long period of time offering thus the opportunity to observe the differences in the clinical course.

Chronic myelogenous leukemia (CML) is a clonal stem cell disorder characterized by the presence of the Philadelphia (Ph) chromosome resulting from the reciprocal translocation between chromosomes 9 and 22, t(9;22)(q34;q11). The translocation leads to the expression of the *BCR-ABL* fusion gene, which is responsible for neoplastic hemopoiesis. In most patients with CML the chromosomal breakpoint in the *ΒCR* gene is located within the major breakpoint cluster region, resulting in two fusion transcripts e13a2 or e14a2 encoding for a 210kDa protein. In rare cases the chromosomal break occurs within the minor breakpoint region and leads to the e1a2 transcript encoding for a 190 kDa protein [1].

The identification of the fusion oncogene *BCR-ABL,* resulted in the development of Tyrosine Kinase Inhibitors (TKIs) giving rise in a new era regarding the treatment of CML. Despite the advances that have been achieved in defining translocation-related oncogenesis, the primary molecular mechanism leading to genetic instability and resulting in *BCR/ABL*-positive CML remains to be defined. However, in contrast to other hematological malignancies, the reports of familial occurrences of CML are extremely rare and therefore a heritable genetic susceptibility has never been established.

In March 2004 a 56- year- old man of Greek origin was admitted to our Department complaining about abdominal discomfort and fatigue. Clinical examination revealed splenomegaly and blood count showed: White Blood Cells 69.800/μL (neutrophils 32%, lymphocytes 9% please change 9% to 18%, eosinophils 8%, basophils 3%, metamyelocytes 21%, myelocytes 11%, promyelocytes 7%), Hemoglobin 11gr/dl and Platelets 674000/μL. Bone marrow aspiration demonstrated extreme hypercellularity with blasts 4% and conventional cytogenetics revealed karyotype 46,XY, t(9;22)(q34;q11) confirming thus the diagnosis of CML in chronic phase, while RT-PCR analysis was positive for e13a2 *BCR-ABL* fusion transcript. He was treated with Imatinib 400mg/day and he reached complete cytogenetic response (CCyR) and major molecular response (MMR) without any indications of resistance. In 2017 he was switched to nilotinib due to bilateral Sensorineural Hearing Impairment probably associated with Imatinib, however, after starting nilotinib he experienced anemia (hemoglobin: 11.4 gr/dL) which compromised his quality of life. Considering that the patient was in Deep Molecular Response (DMR) at the level of MR [4,5] for 6 years, in December 2018 we discussed with him the possibility of treatment discontinuation. Since he met all criteria of therapy discontinuation [2], nilotinib was suspended and he was monitored monthly for the next 6 months, every two months for 6-12 months and every 3 months thereafter, according to discontinuation guidelines [2]. He currently remains in MR [4,5] enjoying a Treatment Free Remission (TFR) of 24 months accompanied by Hb normalization.

In March 2008 the 63-year-old brother of our first patient was diagnosed in another institution with CML in chronic phase. The results of cytogenetic and molecular analysis showed 45,X,-Y t(9; 22)(q34;q11.2) and e1a2 *BCR-ABL* fusion transcript respectively. The patient was treated with Imatinib 400 mg/day. For unknown reasons he was monitored only by qRT-PCR without any preceding confirmation of cytogenetic response. According to the results of qRT-PCR in February 2009 MMR was achieved but in June 2009 loss of MMR was observed and a cytogenetic analysis was performed revealing the presence of Ph chromosome 45,X, -Y t(9;22)(q34;q11.2) in 100% of metaphases. The patient was evaluated in our institution in November 2009. Blood count showed slightly decreased hemoglobin (Hb) (12.1 gr/dL). White blood cells and platelets were normal. A  *BCR-ABL* KD-mutation analysis was performed revealing the presence of F382L KD- mutation. He started treatment with dasatinib 100 mg/day however, he failed to achieve a cytogenetic response, 6 months after treatment initiation. Dasatinib was discontinued due to the development of pleural effusions and therapy with nilotinib 800 mg daily was started, again without achieving any cytogenetic response. Considering that none of the previous treatments led to cytogenetic response, in January 2020 he switched to ponatinib 45 mg daily. After six months of therapy he reached partial cytogenetic response indicating sensitivity to ponatinib.

The primary pathogenic events leading to the Ph chromosome generation and eventually to the development of CML have not been elucidated. Our knowledge about the contributory factors for the development of CML is insufficient. Besides radiation, obesity, adulthood weight gain and cigarette smoking were identified as plausible factors for the development of CML [3,4].

This is the fifth reported case of CML diagnosis among siblings. Tokuhata et al. reported in 1968 the simultaneous diagnosis of CML in a pair of 64-year-old identical twins and one of their siblings, aged 68 years [5]. Philadelphia chromosome was detected in all three patients. Although the twins were symptomatic, the brother had no symptoms and was assessed and diagnosed within 3 months after the occurrence of the disease in the twins.

In 2000 Kapsali et al reported on two siblings diagnosed with CML seven years apart [6]. The first patient, a 47-year-old man was diagnosed only using cytogenetics whereas the diagnosis of his sister was confirmed by both cytogenetics and RT-PCR. Both patients were treated with interferon-A and hydroxyurea. The first patient achieved CCyR, however 6 years after diagnosis, he developed blast crisis and died due to sepsis. His 62-year old sister achieved a hematological remission one year after treatment initiation.

An interesting case of two brothers diagnosed with CML 26 years apart was reported by Lessen et al in 2005 [7]. A 45-year-old man was diagnosed in 2003 suffering from CML by conventional cytogenetics and I-FISH analysis confirmed the presence of *BCR-ABL* gene. The patient was treated with imatinib 400 mg daily. At 6-month and 18-month follow up, he had CCyR. According to obtained medical records his brother was treated for typical chronic phase CML in 1977 and eventually died in 1981 during blast crisis. Although biopsy material was obtained and I-FISH was performed the presence of *BCR-ABL* gene could not be confirmed due to lack of hybridization.

Finally in 2009 Caocci described the occurrence of CML in a female patient and her brother [8]. The 45- aged female patient was diagnosed in 1999 with CML in chronic phase. Cytogenetic analysis showed 46,XX,t(9;22)(q34;q11),i(17)(q10) and RT-PCR revealed the presence of the e13a2 *BCR-ABL* chimeric transcript. The patient was treated initially with hydroxyurea and later on was switched to interferon A combined with subcutaneous Cytarabine and after three months of treatment no hematologic response was achieved. Six months after the diagnosis of CML unrelated bone marrow transplantation was performed and the patient died without achieving engraftment. In 2007, her 50-year-old brother was diagnosed with CML. Cytogenetic analysis and RT-PCR revealed the presence of Ph-chromosome and e13a2 *BCR-ABL* transcript respectively. The patient was treated with Imatinib 400 mg/day and CCyR was achieved and retained after 5 and 20 months of treatment respectively.

Besides the occurrence of CML among siblings, this disease has been also reported occurring in a family, which had at least two affected members. In 1984 Lillicrap et al reported on a 54 year old mother suffering Ph-positive CML and a 14–aged daughter dying at blast crisis two years after the diagnosis of Ph-negative CML [9]. Interestingly the father of the first patient suffered splenomedullary leukemia but no cytogenetic analysis was ever performed.

In a study published by Landgren et al it was demonstrated that relatives of patients with myeloproliferative neoplasms (MPNs), including polycythemia vera, essential thrombocythemia, myelofibrosis and unclassifiable MPN, had significantly increased risks of the above mentioned MPNs. Interestingly, relatives of MPN patients showed also a borderline increased risk of CML [10]. Moreover several studies investigated a potential genetic predisposition to the development of CML based on the presence of certain HLA Class I or Class II molecules. However the underlying mechanisms by which the HLA system might influence susceptibility CML remain unclear.

Importantly, our case of CML occurrence among siblings is the second one investigated by both, conventional cytogenetics and RT-PCR analysis (Table 1). Interestingly although Ph chromosome was detected in both our patients, RT-PCR revealed the presence of two different *BCR-ABL* transcripts. The incidence of CML expressing only e1a2 transcripts is about 1%. Patients with p190 *BCR-ABL* appear to have an inferior outcome after treatment with TKIs. This was also the case of our second patient who showed resistance to multiple TKIs. Unfortunately our patient is ineligible for allogeneic stem cell transplantation due to advanced age. However his prolonged survival underlines the fact that TKI treatment should be continued among chronic phase patients who do not exhibit any cytogenetic response since TKIs seem to confer a survival advantage even in these patients [2]. Moreover the diagnosis of CML in the 2 cases described herein, is of special interest because it is accompanied by a long follow up period. In conclusion we describe a unique case of two brothers suffering from CML based on different fusion transcripts and demonstrating a diametrically opposite course of disease.

**Table 1:** Reports of CML occurring in first-degree relatives.

Authors	No of patients	Age at diagnosis	Relationship	Cytogenetics	RT-PCR
Tokuhata et al	3	64,64,68	Twins,brother	All Ph+	Unknown
Lillicrap et al	3	14,54,69	daughter, mother, grandfather	daughter Ph-, mother Ph+, grandfather unknown	Unknown
Kapsali et al	2	47,62	brother, sister	brother Ph+, sister Ph-	brother unknown, sister RT-PCR+(transcript not provided)
Lessen et al	2	45,30	brother, brother	brother Ph+, brother Ph+	brother unknown, brother RT-PCR + (transcript not provided)
Gaocci et al	2	45,50	Sister, brother	sister Ph+ brother Ph+	sister RT-PCR+(e13a2) brother RT-PCR +(e13a2)
This report	2	56,63	brother, brother	brother Ph+, brother Ph+	brother RT-PCR+(e13a2) brother RT-PCR +(e1a2)

Familial CML may be associated with an environmental factor, with a heritable aspect or both. A possible etiological model might combine the effect of an environmental factor on siblings sharing a genetic predisposition for chromosomal defect. Although the diagnosis of CML in our patients could be coincidental, we believe that our cases emphasize the necessity for collecting and investigating familial CML cases in order to understand the underlying contributory mechanisms in the pathogenesis of CML.
